# Multifaceted Small Molecules as Enzyme Modulators: Cases of Drug Discovery/Repurposing Illustrating Nature′s Pragmatism

**DOI:** 10.1155/bmri/3429646

**Published:** 2026-03-23

**Authors:** Monika I. Konaklieva, Balbina J. Plotkin

**Affiliations:** ^1^ Department of Chemistry, American University, Washington, DC, USA, american.edu; ^2^ Department of Microbiology and Immunology, Midwestern University, Downers Grove, Illinois, USA, midwestern.edu

## Abstract

The cross‐reactivity of substrates, modulators with other enzymes significantly reduces/prevents our ability to design such highly specific, that is, “one warhead–one target”, modulators. On the other hand, the potential “impasse” fuels repurposing of already developed drugs. Additionally, expanding our understanding that there will be “off‐target” effects for enzymes of different evolutionary kingdoms propels the development of covalent reversible drugs. In this review/perspective, we examine these challenges and opportunities based on covalent drugs used/developed for targeting bacterial and mammalian enzymes, and our evolving understanding of the blurred difference between these enzymes in these “vastly” separated organisms by biological evolution.

## 1. Introduction

Enzymes are involved in nearly every biological process, which makes them among the most attractive molecular targets in drug discovery. A challenging aspect of using enzymes as targets is that they can have multiple substrates and functions, a finding that reworks the traditional view of one enzyme catalyzing one reaction with high specificity [1].

The origin of the thousands of enzymes found in modern organisms can be traced back to a relatively limited set of primordial genes and proteins. Evolution led to enzymes with varying degrees of substrate and functional specificity when these variations were beneficial to the organism. A quickly changing environment requires shifts at the molecular level. These changes manifest as an inherent promiscuity in most of an organism′s enzymes. Gene redundancy also increases the number of enzymes at the cellular level, which in turn leads to adaptive significance in the functionality of an enzyme as demonstrated in cross‐kingdom enzyme conservation [2]. Enzyme sequence and structure alignments between that of pathogens and both human metaproteomes and human microbiome have demonstrated that both pathogen and human drug targets are similar in sequence, function, structure and drug‐binding ability for the 1346 FDA‐approved drugs to date [3]. The numbers of off‐target species reported to be affected by drugs for pathogen infections, shown here in descending order are human targets (2243), those of *Escherichia coli* (1146), *Mycobacterium tuberculosis* (Mtb) (1060), *Clostridioides difficile* (990), and *Staphylococcus aureus* (863). Thus, phylogenetically diverse bacteria could be susceptible to drugs irrespective of the targeted organism [3].

Although a limited number of enzymes that are highly specific to their substrates exist, the evolutionary advantage of fast adaptation through enzyme promiscuity to new/challenging environments is highly advantageous [4]. This is particularly important since it leads to enhanced fitness, such as the development of resistance by bacteria to *β*‐lactam antibiotics in response to the environmental pressure of current treatments of infectious diseases [5]. For example, the pressure of *β*‐lactam antibiotics has driven the broadening of the hydrolytic profile of the *β*‐lactamases [5]. However, even with the expansion of these enzymes′ classes that support highly diverse chemistry on many different substrates, nature utilizes a limited number of protein folds in the chemical space. Therefore, a major challenge in the design of enzyme modulators is achieving selectivity and specificity in order to limit off‐target effects [6].

Although toxicity is always a concern for the off‐target effects of covalent modulators [7], recent exploration of predominantly antimicrobial covalent small molecules demonstrates a different perspective into interkingdom enzyme modulation [8]. One advantage of enzyme promiscuity is that target multiplicity offers the opportunity for drug off‐label use, that is, drug repurposing. For less complex diseases with well‐established mechanisms, the use of a single drug for a single target has been successful for treatment management [9–11]. However, for complex and multifactorial diseases, including cancers, infectious diseases, and central nervous system (CNS) disorders, the single drug target approach has become ineffective mainly as a result of the development of drug resistance [9–11]. Thus, for complex diseases, a polypharmacologic drug that modulates multiple enzymes/pathways involved in disease onset and development is needed [11]. Interestingly, retrospective examination of several therapeutically efficacious drugs revealed that they modulate multiple molecular targets [12]. These observations have fueled the push over the latest quarter of a century toward multitarget drug discovery as a highly attractive alternative to the traditionally dominant single‐target approach [13–15].

This review examines the inherent challenges in developing specific enzyme modulators. Examples of clinically relevant small molecules, natural or synthetic, that possess the appropriate electrophilic moiety to act as cross‐inhibitors of target domains for either prokaryotic or eukaryotic targets are discussed. The enzyme modulators discussed herein are covalent drugs (enzyme inhibitors), and their introduction is in the chronological order of their utilization from natural products (irreversible) to current trends (irreversible/reversible) based on nature‐inspired warheads.

**Figure 1:**
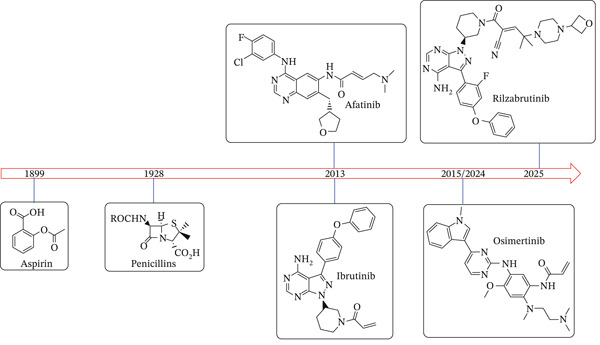
Timeline of development of major covalent drugs discussed herein; including the first FDA‐approved reversible cyanoacrylamide‐containing warhead rilzabrutinib.

Covalent enzyme inhibitors can offer significant advantages over noncovalent inhibitors, since they possess a warhead that could target a single amino acid residue in a given protein target. We have chosen two different drug classes, which are currently at the two ends of the drug discovery spectrum, that is, *β*‐lactam antibiotics (as acylating agents) and kinase inhibitors, specifically those that act as Michael acceptors (Figure 1). Michael acceptors are electrophilic molecules (more specifically containing an *α*,*β*‐unsaturated electrophilic center) that have a greater affinity to bind to cysteine as compared with serine, as well as to lysine or histidine enzymes.

*β*‐Lactam antibiotics, which are natural products that were discovered in 1928, represent the second covalent drug class, after aspirin, to become clinically relevant. Historically, they are well known as inhibitors of bacterial serine proteases, which are associated with the building and integrity of the bacterial cell wall. Irreversible Michael acceptors as biologically active compounds exist in nature, predominantly as plant metabolites, whereas the first irreversible synthetic kinase inhibitors only received FDA approval about a decade ago (2013). *β*‐Lactams remain widely used antibiotics due to their generally safe profile for humans and because they enjoy excellent pharmacodynamic and pharmacokinetic profiles. To prolong their clinical relevance in the current era of microbial resistance, strategies to evade/block the mechanisms through which bacteria sense and neutralize the antibiotics′ action are being developed. One drug class that offers a synergistic opportunity for prolonging the life of these antibiotics is human kinase inhibitors, which are currently approved for the treatment of different cancers.

Kinases are one of the largest families of human enzymes [16]. Kinases mediate cell signal transduction by catalyzing the transfer of the ATP phosphate to various specific substrates [17]. These phosphorylated substrates control numerous important biological functions, including cell proliferation and apoptosis, which makes them targets for anticancer drugs. The development of kinase inhibitors in the last decade was one of the fastest‐growing areas of drug development, with this year (2025) marking the FDA approval of the 100th small kinase inhibitor. This is fairly remarkable since kinases were considered to be undruggable targets as late as the 1990s. Recently, kinase inhibitors have been expanding into immunological targets associated with rheumatoid arthritis, psoriatic arthritis, atopic dermatitis, and ulcerative colitis [18]. Overall, more than 150 kinase‐targeted therapies are currently undergoing clinical trials or are in preclinical stages of development [18, 19].

Most FDA‐approved kinase inhibitors can bind to multiple kinases. Fostamatinib (Tavalisse, Rigel Pharmaceuticals, known as “SYK inhibitor”), being the extreme example, by inhibiting more than 250 kinases [18]. This promiscuity leads to a negative side effect for nonspecific kinase inhibition—predominantly cardiotoxicity. Furthermore, greater than 20,000 proteins that are not kinases could potentially be inhibited by the currently identified/FDA‐approved kinase inhibitors [19]. This toxicity, or additional modes of kinase inhibitors′ action, is summarized in a recent excellent review [19]. Several examples of the activity of the kinase inhibitors on nonkinase targets in humans are mentioned herein [20, 21]. Conversely, the development of dual inhibitors, including those having an *α*, *β*‐unsaturated (acrylate) warhead, which secures covalent binding, has been developed to tackle the challenge presented by the multitarget, multisignaling pathways involved in cancer onset and progression. Such dual inhibitors include drugs with activity against anaplastic lymphoma kinase (ALK) and epidermal growth factor receptor (EGFR), which is used to treat non‐small cell lung cancer (NSCLC), as well as by MER/AXL (receptor tyrosine kinase [RTK]) in the TAM family (TYRO3, AXL, MER), which is overexpressed in various types of hematological and solid tumor cancers [22–24].

The molecular targets of the human kinase inhibitors are nucleophilic residues such as cysteine, lysine, and tyrosine that are in proximity to, or within the binding site of their respective kinases. The preferred target of the kinase inhibitor is the noncatalytic cysteine, especially those inhibitors with Michael acceptors as the warhead, due to the cysteine′s intrinsic nucleophilicity. The recent advances in the development and clinical trials of the covalent kinase inhibitors are nicely reviewed [18, 25].

In this review, the utilization of kinase inhibitors in host‐directed therapy against infectious diseases, as well as human oncological and non‐oncological therapies, is discussed with a focus on covalent inhibitors, and a specific emphasis on the advances in reversible covalent inhibitors (Michael acceptors, Figure 1). In addition, we provide further perspective on the advantages of reversible covalent kinase inhibitors (RCKIs) over their irreversible counterparts.

## 2. Methodology

For this review, a selective approach was used to identify relevant scholarly publications. This examination of the literature focused on the inherited challenge in the design of selective enzyme modulators. Only articles published in English were considered for inclusion using all major academic databases and search engines, such as SciFinder, Reaxys, Google Scholar, and PubMed. The articles included in this review, both foundational and contemporary studies, spanning from 1971 to 2025. Of the 144 included references, 97 articles (67%) were published within the last decade (2015–2025), indicating the intensity of research in the area of polypharmacology in the last 25 years and specifically in the last decade. Key words such as “enzyme specificity,” “enzyme promiscuity,” “covalent inhibitors,” “*β*‐lactams,” “kinase inhibitors,” “drug promiscuity,” “polypharmacology,” and “pathogen‐targeted therapy” were used to identify relevant publications. Abstracts were analyzed, followed by full‐text reviews of those with relevance to the topic of this review.

## 3. Covalent Irreversible Clinically Relevant Drugs With Multiple Bacterial/Viral/Mammalian Targets

### 3.1. Acylating Agents

Penicillin and aspirin (acetylsalicylic acid) are established acylating agents. Both drugs have antimicrobial activity, with the *β*‐lactams being the most extensively studied and shown to be inhibitors of the bacterial penicillin‐binding proteins (PBPs) [26]. All PBPs contain active‐site serine residues that can be acylated by penicillin, resulting in the inhibition of PBP activity and leading to cell membrane rupture [26]. Although the molecular mechanism of aspirin′s antimicrobial activity has not been delineated, its anti‐inflammatory action is accomplished by acylating Ser529 in the substrate‐binding channel of mammalian cyclooxygenase 1, thus preventing the conversion of the substrate arachidonic acid into prostaglandins [27, 28]. There is now compelling evidence that representatives of *β*‐lactam antibiotics are involved in the modulation of mammalian immunity, and aspirin—in the modulation of bacterial/viral enzymes.

#### 3.1.1. *β*‐Lactam Antibiotics as Modulators of Mammalian Host Immunity

The mammalian host invaded by bacteria activates a broad range of innate immune responses upon sensing the presence of bacteria. Pathogen sensing can be achieved by pattern recognition receptors (PRRs), which are highly conserved throughout vertebrate evolution. These receptors are expressed at the cell surface, as well as within the cell, on the endosome membranes, and in the cytoplasm. The presence of the PRPs in these locations secures the detection of both extra‐, as well as intracellular pathogens. This detection is achieved mainly by recognition of the pathogens′ nucleic acids (bacterial and viral), lipopolysaccharides (LPSs), lipoteichoic acids (LTAs), and carbohydrates. *β*‐lactam activity on bacteria can result in the release of pathogen‐associated molecular patterns (PAMPs), which in turn could affect the anti‐infective response of the host′s myeloid cells. These cells are the first line of defense against bacterial infections and include monocytes, macrophages, neutrophils, and dendritic cells. Therefore, a *β*‐lactam antibiotic can have activity beyond bactericidal, such as direct immune cell binding and immune cell modulation via initiating the release of bacterial PAMP/toxins [29–31].

PAMPs are required for a microbe′s essential functions, thus they exhibit a high degree of organism specificity [29–31]. This results in diverse microorganisms being affected differently by the representatives of the *β*‐lactam class of antibiotics. Several examples are given below.

- LTA and peptidoglycan fragments′ production is increased in Gram‐positive bacteria upon treatment with *β*‐lactam antibiotics, more specifically penicillins **1** (Table 1). When penicillin (**1a**, Table 1) or oxacillin **(1b,** Table 1) are used against *Staphylococcus epidermidis,* the increased release of LTA results in the production of tumor necrosis factor (TNF) by the host′s immune cells [32, 33].
- LPS (endotoxin), a molecule found in the outer membrane of Gram‐negative bacteria, can cause hypovolemic shock [31]. The type of *β*‐lactam antibiotic and its concentration can differentially induce different amounts of LPS release. Cephalosporins **2** (Table 1), which target PBP‐3, induce a higher level of LPS release as compared with carbapenems, whose molecular target is the PBP‐2. Furthermore, the cephalosporin effect is also concentration dependent—with subMIC (minimal inhibitory concentration) drug levels eliciting a greater release of LPS by the Gram‐negative bacteria [34, 35].
- The production of pneumolysin, a pore‐forming toxin and major virulence factor of *Streptococcus pneumoniae*, is enhanced upon exposure to cephalosporins **2**, (Table 1) specifically ceftriaxone (**2a**, Table 1) [36, 37].

**Table 1:** Activity of penicillins and cephalosporins, beyond their intrinsic antibacterial activity.

Structure/main activity (known molecular target)	Molecular target of the “Off‐target” activity/MOA	“Off‐target” activity/references
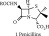 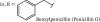 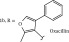 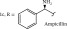 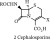	Specific target/MOA unknown. [32, 33]	Lipoteichoic acid (LTA), and peptidoglycan fragments′ production is increased in Gram‐positive bacteria [32, 33]
Inhibitors of PBPs–bacterial serine proteases [26] 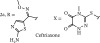 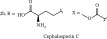 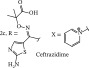	Specific target/MOA unknown. [34, 35]	Enhanced production of pneumolysin (ceftriaxone); enhanced production of bacterial membrane vesicles (*Acinetobacter baumannii)* and cross‐protection for *Pseudomonas aeruginosa* and *Streptococcus pneumoniae by Stenotrophomonas maltophilia* [34–39]
A *β*‐lactamase inhibitor 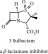 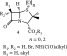	Inhibitor of bacterial and human *β*‐lactamases. [40] Inhibitors of M^pro^ COVID‐19 viral cysteine protease. [41, 42]	Inhibits MBLAC2, a human *β*‐lactamase [40]Antivirals against COVID‐19 [41, 42]

Almost all bacteria produce bacterial membrane vesicles (MVs). These MV contain a variety of PAMPs that are involved in virulence, bacterial resistance to antibiotics and host immunity [38–43]. Drug‐induced MVs elicit a pro‐inflammatory response in animal models (mice), which can result in an increased resistance to heterologous infections [43]. Although the bacterial responses elicited by the action of the *β*‐lactam antibiotics, which subsequently trigger the host′s immune responses, are expected, the direct action of the *β*‐lactams on the host′s immune cells is a new feature in our understanding of the effect of the *β*‐lactams on mammalian immunity. Although the mechanism(s) by which *β*‐lactams directly modulate the host′s immune responses have not, as yet, been elucidated, findings show that macrophage exposure to ceftriaxone (**2a**, Table 1) enhances the expression of the proinflammatory cytokines IL‐1beta and TNF‐*α*. In addition, mouse cells are more permissive for *Salmonella enterica* serovar Typhimurium adherence [44].

In another example, ceftriaxone (**2a**, Table 1) and ceftrazidime (**2c**, Table 1) stimulate phagocytosis of macrophages and neutrophils both in vitro and in vivo [45] presumably due to increased reactive oxygen species (ROS) production [46].

With regards to bacterial response in general, production of MVs in response to lactams can be highly variable. Cephalosporin 2b (Table 1) elicits enhanced production of MVs by *Acinetobacter baumannii*, whereas carbapenems have no such effect [38]. In addition, MVs′ induction of drug‐resistance can be complex, with a response to *β*‐lactams resulting in cross‐protection between bacterial species. For example, upon exposure to *β*‐lactams, *Stenotrophomonas maltophilia* releases MVs containing *β*‐lactamases [39]. These MVs in turn protect antibiotic‐susceptible *Pseudomonas aeruginosa* and *S. pneumoniae* from *β*‐lactam antimicrobial activity [45].

The high level of bacterial resistance toward this drug class is mediated by the production of *β*‐lactamases. Inhibitors of bacterial *β*‐lactamases from both the serine‐ and metallo‐*β*‐lactamase classes have been discovered. A combination of a *β*‐lactam antibiotic/serine *β*‐lactamase inhibitor is an FDA‐approved drug. The identification to date of 18 human metallo‐*β*‐lactamases (hMBLs) containing either Zn^2+^ or Fe^3+^ or Mn^2+^ as the Lewis acid in their active sites is interesting, but not entirely surprising [47, 48]. Of the currently known hMBLs, all have very high similarity with the conserved active site “motifs” present in the prokaryotic MBLs (the *β*‐lactam hydrolysing enzymes) [48]. However, the majority of reactions/substrates they are associated with differ from their *β*‐lactam–hydrolyzing prokaryotic ancestors. Based on phylogenetic analyses, hMBLs are placed in three groups: Group 1 (seven enzymes) represents glyoxalase II family‐related enzymes. Although most of the functions of its seven members have not been clearly defined, the functions of two members of this group have been identified. They include detoxification of reactive and toxic 2‐oxoaldehydes (HAGH MBl) and the mitochondrial metabolism of the toxic H_2_S (ETHE1 MBL). The latter enzyme is overexpressed in hepatocellular carcinoma cells and is proposed to negate apoptosis, which leads to enhanced cancer progression [48]. The Group 2 MBLs (nine enzymes) are involved in nucleic acid modulation, with some linked to anticancer drug resistance [48]. The enzymatic role of most of them is currently unknown; however, mutations in some hMBLs have been linked to several immunological diseases [40, 47–49]. One of the well‐established hMBLs mechanisms is that of the SNM1A, B, and C proteins, which are involved in DNA repair. Cephalosporins are competitive inhibitors of the hMBLs SNM1A, B, and C nuclease [50]. Ampicillin (**1c**, Table 1) and cephalosporin (**2b**, Table 1) inhibit in vitro SNMIC/Artemis protein [51, 52]. The SNM1C/Artemis protein is associated with the V(D)J segments rearrangement, which leads to immunoglobulin and T cell receptor variable regions; thus, its highly important role in the immune response. Since the conserved catalytic site is highly similar in SNM1A, B and C proteins, it has been postulated that the *β*‐lactam antibiotics can potentially inhibit the SNM1C/Artemis protein, leading to a transient humoral immune deficit [51, 53]. Similar to their bacterial counterparts, hMBLs also hydrolyse *β*‐lactam antibiotics [40]. Some hMBLs (the DNA crosslink repair enzymes SNM1A and B) enable resistance to anticancer drugs such as mitomycin C and cisplatin in parallel with the role of the prokaryotic MBLs in antibiotic resistance. Human metallo‐*β*‐lactamases (LactB2, MBLAC2, SNM1A, and SNM1B), like those found in bacteria, have been tested for *β*‐lactamase activity [40, 50]. *β*‐lactam hydrolysis using nitrocefin was detected for the MBLAC2 enzyme as well as for penicillin G (**1a**, Table 1) [40, 50]. Sulbactam (**3**, Table 1), a *β*‐lactamase inhibitor, inhibits the direct effect of penicillin on human cells in the MBLAC2 enzyme [40]. For additional, in‐depth information on the host immune responses to the *β*‐lactam antibiotics, as well as the utility of *β*‐lactams as anticancer drugs, the reader is directed to these excellent reviews [8, 54, 55].

Group 3 consists of hMBLs (NAPE‐PLD and CMAH) which exhibit more diverse functions. N‐Acyl‐phosphatidylethanolamine‐hydrolyzing phospholipase D (NAPE‐PLD) plays an important role in the conversion of metabolic lipids into signaling molecules, for example, neurotransmitter N‐arachidonoylethanolamine (anandamide), that binds to cannabinoid receptors [48]. Cytidine monophospho‐N‐acetylneuraminic acid hydroxylase (CMAH) catalyzes the addition of an oxygen atom onto the methyl group of the acetyl group of CMP‐Neu5Ac to give Neu5Gc. The latter is a common form of sialic acids, which are attached to the sugar chains on the surface‐exposed portion of plasma membrane proteins. Sialic acids play important roles in ligand–receptor, cell–cell, and cell–pathogen interactions. Catalytically active CMAH is present in all mammals, with the exception of humans [48]. The lack of catalytic activity of CMAH in humans, which occurred before the Neanderthal divergence, is proposed to be associated with the acquisition of resistance/susceptibility to pathogens that preferentially recognize Neu5Gc or Neu5Ac. The catalytically inactive human “CMAH‐like” protein has involvement in glucose metabolism. CMAH−/− knockout mice exhibit an obese phenotype with impaired glucose tolerance and pancreatic B cell dysfunction [48].

Although lactam antibiotics are best known as serine protease inhibitors, they can inhibit cysteine enzymes. An illustrative example is the inhibition of the main viral protease (M^pro^) of SARS‐CoV‐2, a cysteine hydrolase, and the current target for antiviral chemotherapy by *β*‐lactams, including penicillin esters (**4**, Table 1), acting as acylating agents for M^pro^ inhibition [41, 42].

#### 3.1.2. Aspirin

Aspirin (acetylsalicylic acid, Figure 1), in addition to its anti‐inflammatory effects, can also inhibit bacterial growth [56]. The antimicrobial properties of aspirin have been investigated for the past 2 decades and are elegantly reviewed [57]. However, the results of the antibacterial/immunological activity are microbe‐specific. Some reports indicate that growth in the presence of aspirin of certain bacteria induces an intrinsic multiple antibiotic‐resistant phenotype. In other bacterial species a reduction in the resistance of bacteria to some antibiotics is observed [57]. These “conflicting” results are most likely due to the ability of this small molecule to bind to a multitude of both prokaryotic and eukaryotic targets, with different nucleophilic residues in its active site (e.g., serine/cysteine enzymes), most of which have not been clearly defined. An illustration for that is a bacterial enzyme that aspirin has been shown to inhibit, arylamine N‐acetyltransferase (NAT) in *Klebsiella pneumoniae* [58]. NAT, a cysteine enzyme, catalyzes the transfer of acetyl groups from acetyl‐CoA to arylamines, and it is involved in several metabolic functions, including the inactivation of drugs. This cysteine forms part of an active‐site catalytic triad composed of Cys‐His‐Asp that is conserved in all prokaryotic and eukaryotic NAT homologs, which is a prerequisite for at least some of the aspirin targets [59]. Recent studies have demonstrated aspirin′s anticancer properties. These properties include those that target colorectal adenoma [60–62]. In addition, it has synergistic activity with vascular endothelial growth factor (VEGF) and platelet‐derived growth factor (PDGF) receptors inhibitors, such as lenvatinib, which have been widely adopted in cancer treatment, despite this synergistic approach risking the development of aortic dissection (AD) in cancer patients.

## 4. Kinase Inhibitors Restore the Activity of *β*‐Lactams Against Bacteria

Different bacterial kinases are found in many important pathogens. For example, *S. aureus*, *Listeria monocytogenes*, Mtb, and *Enterococcus faecalis* express a bipartite membrane‐associated eukaryote‐like serine/threonine kinase (eSTK). This family of enzymes is known as the penicillin‐binding protein and serine/threonine kinase‐associated protein (PASTA) kinases [63]. PASTA kinases have extracellular penicillin‐binding domains involved in binding fragments of peptidoglycan produced as a result of cell wall damage/remodeling and an intracellular serine/threonine kinase domain similar to those of eukaryotic cells [64]. Although the substrates and functions of the PASTA kinases are still being defined, it appears that their function is microbe‐specific. In *Streptococcus mutans*, they are involved in biofilm formation, whereas in Mtb, they appear to be essential enzymes [65–68]. The latter has fueled a novel antimicrobial therapeutic strategy against Mtb by inhibiting Mtb kinase PknB, a PASTA kinase [67, 69–71]. A similar strategy is being directed against bacteria, including methicillin‐resistant *Staphylococcus aureus* (MRSA), based on the fact that the deletion of their respective PASTA kinase sensitizes them to certain *β*‐lactam antibiotic activity [72–75].

More recently, a bacterial membrane‐associated histidine kinase has been reported that functions as an alternative trigger for *β*‐lactamase production [76]. This signaling system directly detects *β*‐lactams, providing a rapid response to the presence of *β*‐lactam antibiotics without the need for *β*‐lactam–induced cell wall damage. Interaction of *β*‐lactam antibiotics with the histidine kinase results in the immediate production of *β*‐lactamases, providing a prompt defense against the antibiotics.

### 4.1. Kinase Inhibitors—Focus on Michael Acceptors

Several of the reported inhibitors of bacterial histidine kinases contain a Michael acceptor moiety, as well as a cyano group [77, 78]. Although not all the modes of action (MOA) of these inhibitors are elucidated to date, they have the potential to bind as Michael acceptors or via the cyano group to these enzymes′ cysteines.

#### 4.1.1. Host‐Directed Therapies (HDTs)

##### 4.1.1.1. Antimicrobials.

Bacterial pathogens, for example, *Salmonella* and Mtb, and viruses depend on human host′s enzymes for their survival and proliferation. Unfortunately, microbial resistance makes disease treatment challenging. One of the approaches that currently is being explored is HDT. Compounds that target host RTKs signaling and inhibit intracellular MDR Mtb *and Salmonella* have been identified by using a library of pharmacologically active compounds (LOPAC)–based drug‐repurposing screen. In addition, a human kinome siRNA screen independently confirmed the role of RTK signaling and kinases (BLK, ABL1, and NTRK1) in the host control of Mtb [79]. Another study identified two known kinase inhibitors with activity against Mtb and *Salmonella typhimurium*, thus expanding the potential of HDT against these pathogens [80]. Furthermore, an inhibitor of Syk tyrosine kinase was also shown to reduce inflammation in an in vitro model of *P. aeruginosa* infection [81].

##### 4.1.1.2. Antivirals.

Viruses capture a significant number of host kinases during replication; therefore, approved kinase inhibitors from various chemotypes have been repurposed as host‐targeted, broad‐spectrum antiviral therapies [82]. Inhibitors of EGFR and ERBb kinase family ERBB2, erlotinib, gefitinib, and lapatinib demonstrate in vitro and in vivo antiviral activities against hepatitis C virus (HCV) and human cytomegalovirus [83, 84]. The c‐Abl inhibitors, imatinib and nilotinib, inhibit replication of Ebola virus, dengue virus (DENV), and Middle East Respiratory Syndrome coronavirus (MERS‐CoV). In addition, several other kinase inhibitors have demonstrated broad‐spectrum in vitro antiviral activity, including against severe acute respiratory syndrome (SARS)‐CoV and influenza viruses [85–94].

## 5. Irreversible Michael Acceptors

The Michael acceptor‐containing ibrutinib (**5**, Table 2) developed as an inhibitor of Bruton′s tyrosine kinase (BTK) (FDA approved, 2013) appears to also have a direct effect on the mammalian response to infection, despite its lack of intrinsic bacteriocidal/bacteriostatic activity [95–110]. Ibrutinib (**5**, Table 2) belongs to the newer tendency in drug discovery wherein the ligand is targeted first. Ibrutinib has been shown to protect *S. pneumoniae*–infected animals treated with ceftriaxone by decreasing the activation threshold of monocytes–macrophages and neutrophils [95]. Ibrutinib also inhibits inflammatory responses in a model of LTA induced acute lung inflammation [95, 96, 109, 110]. Ibrutinib demonstrated HDT in antimicrobial activity against intracellular Mtb growth by inducing macrophage autophagy [98]. To date, although the mechanism of action on immune cells is undetermined, ibrutinib has not been shown to have any adverse effects in immunocompromised patients [95, 96].

**Table 2:** Representative examples of FDA‐approved irreversible Michael acceptors that have demonstrated anti‐infective activities in addition to their anticancer activity.

Structure/main activity (known molecular target)	Molecular target of the “Off‐target” activity/MOA	“Off‐target” activity/references
Ibrutinib/FDA appr. 2013Bruton′s tyrosine kinase inhibitor 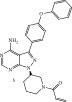 [95]	Specific target/MOA unknown. [95, 96]	Inhibition/decrease of the inflammatory cytokines (toll‐like receptor pathway) in macrophages, which are the cause in the majority of pulmonary injury in COVID‐19 disease [95, 96]
Sunitinib/FDA approved 2006/2017Receptor tyrosine kinase inhibitor 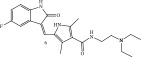 [97]		Broad antiviral properties COVID‐19, Zika [98–100].Antibacterial activity against *E. coli* and *Staphylococcus aureus* [101].
Afatinib/FDA appr. 2013An epidermal growth factor receptor tyrosine kinase (EGFR) inhibitor and erythroblastic leukemia viral oncogene homolog (ErbB) tyrosine kinase inhibitor 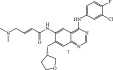 [97, 102]	Specific target/MOA unknown. [97, 102, 103] Multiple steps of the mammarenaviruses’ life cycles. [101]	Bactericidal to biofilm cells. *E. coli, P. aeruginosa* and *Salmonella typhimurium* [97, 102]Antiviral against mammarenaviruses, for example, Lassa fever virus (LASV) and lymphocytic choriomeningitis virus (LCMV), and also against Junin virus. [103]
Dacomitinib/FDA appr. 2018An epidermal growth factor receptor tyrosine kinase (EGFR) inhibitor 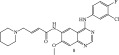 [102]	FtsZ protein (filamentous temperature‐sensitive mutant Z) via hydrophobic and H‐bonding interactions. [104]	Inhibition of bacterial cell division.MICs 16 *μ*g/mL against *Bacillus subtilis* 168 and 32–64 *μ*g/mL against *S. aureus* strains [104] *B. subtilis,* S*. aureus*, MSSA, and MRSA strains. (abbreviations defined in text earlier)Antiviral against COVID‐19. [105–107]
Osimertinib/FDA appr. 2015‐2024FDA approvals for various stages and mutations of non‐small cell lung cancer.EGFR tyrosine kinase inhibitor. 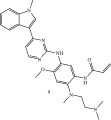 [108]	Blocks the epidermal growth factor receptor (EGFR) pathway. [108]	Antiviral activitySARS‐COVID‐2 variant. [108]

Sunitinib (**6**, Table 2), another tyrosine kinase inhibitor, shows activity against coronavirus disease 2019 (COVID‐19) as well as Zika virus [98–100]. In addition, sunitinib, when incorporated into a hydrogel drug delivery system to increase its hydrophilicity, demonstrated antibacterial activity against *E. coli* and *S. aureus* [101].

Afanitib (**7**, Table 2) is an EGFR tyrosine kinase inhibitor with bactericidal activity against *E. coli, P. aeruginosa*, and *S. typhimurium* in formed biofilms [97, 102]. Furthermore, it has demonstrated antiviral activity against mammarenaviruses [103].

Dacomitinib, (**8** Table 2), which is structurally similar to afatinib, also exhibits antibacterial and antibiofilm activity [102]. Dacomitinib (**8**, Table 2) was found to be a potential inhibitor of the FtsZ protein (filamentous temperature‐sensitive mutant Z), the first protein recruited to the division site in bacterial cells during replication. Dacomitinib exhibits antibacterial activity against *Bacillus subtilis* and methicillin‐sensitive *Staphylococcus aureus* (MSSA) and MRSA strains, with the best antimicrobial activity against MRSA strains [104]. Dacomitinib also has activity against *E. coli*, but only in the presence of compounds that can increase the permeability of the outer membrane [104]. Modifications in dacomitinib (**8**, Table 2) aimed at increasing its water solubility are expected to enhance its antimicrobial activity [104]. In addition, dacomitinib has demonstrated antiviral activity against COVID‐19 [90, 106, 107].

Another compound in this group is osimertinib, (**9**, Table 2) which has a demonstrated reduction in infectivity of highly infectious SARS‐COVID‐2 variant [108].

The full list of the FDA‐approved kinase inhibitors can be found at the Blue Ridge Mountain Medical Research website: https://brimr.org/protein-kinase-inhibitors.

The transcription factor NF‐*κ*B regulates multiple aspects of innate and adaptive immune functions and serves as a pivotal mediator of inflammatory responses. I*κ*B (inhibitor of *κ*B) is a family of NF‐*κ*B negative regulators that bind to NF‐*κ*B, preventing it from activating gene transcription. Both Bay 11‐7082,**10** and Bay 11‐7085, **11** (Table 3) block I*κ*B phosphorylation and degradation, thus maintaining NF‐*κ*B inhibition and subsequently reducing inflammation [111–117]. At 10 *μ*M (2.5 *μ*g/mL), Compound **11** (Table 3) irreversibly inhibits I*κ*B‐*α* phosphorylation, resulting in anti‐inflammatory efficacy in both the rat adjuvant arthritis and carrageenan rat paw edema model systems [111]. In addition to its anti‐inflammatory effects, Compound **11** (Table 3) also induces cancer cell apoptosis through inhibition of NF‐*κ*B signaling. Moreover, it has NF‐*κ*B–independent anti‐cancer activity [112].

**Table 3:** Irreversible Michael acceptors—currently for research only, that have demonstrated anti‐infective activities in addition to their anti‐inflammatory/anticancer activity.

Structure/main activity (known molecular target)	Molecular target of the “Off‐target” activity/MOA	“Off‐target” activity/references
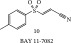 I*κ*B*α* phosphorylation and NF‐*κ*B inhibitor.I*κ*B kinase and protein tyrosine phosphatases (PTPs) active site inhibitor. [111, 112] 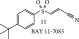	MOA undetermined [113]	Bactericidal alone and in combination Compound 13 adjunctive for penicillin G. (16‐fold reduction in MIC). [113]
TNF‐*α*–stimulated I*κ*B*α* phosphorylation inhibitor. [111, 112] 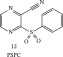 [111] 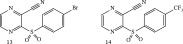 [113, 114]	Specific target/MOA unknown. [115] MOA undetermined [94 = 114]	Antibiofilm as monoculture and polymicrobial coculture. *S. aureus* (MRSA—4 *μ*g/mL) and *Candida albicans*/antibiofilm. [115] *S. aureus*, MSSA, *C. albicans.* Antiviral against African swine fever virus (ASFV). [116, 117] For Compounds 13 and 14, the reduction of penicillin G MIC to 0.39 *μ*M. *S. aureus* MRSA modest antimicrobial activity against Gram‐negative, for example, *P. aeruginosa.* [113, 114]

Bay 11‐7082 (**10**, Table 3) has been proposed to have, in addition to the aforementioned activities of Bay 11‐7085 (**11**, Table 3) inhibitor activity against mammalian protein tyrosine phosphatases (PTPs), which regulate various cellular processes [118]. Furthermore, it has demonstrated activity against *Candida* species, even though the fungus lacks intrinsic NF‐*κ*B signaling [115]. In addition, Bay 11‐7085 (**11**, Table 3) demonstrated in vitro and in vivo bactericidal activity against MDR *S. aureus* with a MIC of 4 *μ*g/mL [115]. Moreover, in biofilm studies, Bay 11‐7085 (**11**, Table 3) inhibited *S. aureus*–*Candida* spp. polymicrobial biofilm formation [115].

Due to its antistaphylococcal activity, Bay 11‐7082 (**10**, Table 3) medicinal chemistry protocols were used toward structure optimization [113]. From the library of compounds synthesized which eliminated its Michael acceptor activity, only the replacement of the tert‐butyl group with a F‐atom on the phenyl ring retained the inhibitory activity of Bay 11‐7082 (**10**, Table 3) toward *S. aureus*. Further synthetic efforts were directed toward replacing the phenyl ring with a pyrazine ring (Compounds **12–14,** Table 3) while retaining the cyano‐group, but not as part of the *α*, *β* ‐unsaturated system. The latter was an attempt to reduce the possible off‐target binding (toxicity) of the original Compound **10**, Table 3 [113]. The preparation of these latter compounds (**12–14**, Table 3) was inspired also by the earlier report for sulfone **12** (Table 3) having antistaphylococcal activity [114]. Of these, Compounds **13** and **14** (Table 3) are the most promising compounds as adjuvants for Penicillin G (Pen G) since they can potentiate Pen G activity against MRSA, shifting the MIC from 3.74 to 0.39 *μ*M, a value that is comparable with the reduction of the MIC by Bay 11‐7082 (**10**, Table 3, MIC of penicillin G from 3.74 to 0.23 *μ*M, a 16‐fold reduction). The authors assumed that both compounds (**13** and **14**, Table 3) would not act as Michael acceptors [113]. Finally, some viruses, like certain strains of influenza virus, rely on NF‐*κ*B signaling pathways for their replication. By inhibiting NF‐*κ*B, BAY 11‐7085 (**11**, Table 3) can potentially interfere with these viruses′ ability to replicate within host cells [116, 117].

Kinases are pivotal in many human cellular pathways. In addition to kinases involved in various oncological disease states, kinase signaling also plays an indispensable role in all major human diseases such as immunological, inflammatory, degenerative, metabolic, and cardiovascular. Thus far, the JAK inhibitors are the leading inhibitors in the non‐oncological space. These drugs block inflammatory JAK‐STAT signaling, which allows for their utilization/FDA approval for different inflammatory indications, such as rheumatoid arthritis, psoriatic arthritis, atopic dermatitis, and ulcerative colitis. Since BTK signaling also modulates adaptive and innate immune signaling, the potential of these agents in chronic diseases has been explored. Two BTK inhibitors, Compounds **15** and **16** (Table 4), which are representatives of the irreversible Michael acceptor drug class, serve as illustrative examples of their utility in immune disease states [20, 21].

**Table 4:** FDA‐approved/pending review covalent kinase inhibitors for uses other than anticancer therapies.

Structure	Molecular target/activity/MOA	FDA‐approved for
Remibrutinib/FDA‐approved, 2025An oral, selective inhibitor of Bruton′s tyrosine kinase (BTK). 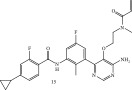	Binds to BTK in its inactive form, suppressing the downstream cascade, and release of histamine and proinflammatory cytokines. [20]	The first kinase inhibitor approved for chronic spontaneous urticaria (CSU) in adults whose symptoms are not adequately controlled by antihistamines.
Tolebrutinib/pending FDA approval decision by the end of 2025. 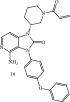 The revised target action date for the FDA decision is December 28, 2025.	BTK inhibitor in the central nervous system (CNS) and peripherally, thus modulates B‐lymphocytes and activated microglia, which are immune cells involved in inflammation and neurodegeneration in diseases like multiple sclerosis (MS). [21]	An oral and brain‐penetrant investigational BTK inhibitor to treat nonrelapsing, secondary progressive multiple sclerosis (nrSPMS) and to slow disability accumulation independent of relapse activity in adult patients.

## 6. Reversible Michael Acceptors

RCKIs are attracting increasing attention. These inhibitors demonstrate selectivity without irreversible protein modification, thus diminishing the inhibitors′ adverse effects. Cyanoacrylamide is the most frequently used electrophilic moiety in the reversible covalent inhibitors, replacing the acrylamide moiety in the irreversible covalent inhibitors [119]. The replacement of warheads is a strategy used to reduce potential toxicity by limiting the reactivity of irreversible covalent inhibitors, in which the inhibitor is considered to be a substrate that is catalyzed by its target enzyme. Several RCKIs are currently undergoing clinical trials as kinase inhibitors, and some are already FDA approved [25, 119–137].

The electrophilic part of the reversible inhibitor, for example, cyanoacrylamide, is attacked by one reachable nucleophile in the enzyme binding site (most often, but not limited to a noncatalytic cysteine), followed by a reversible chemical reaction. The reversible covalent inhibition has two significant properties: It ensures high potency of binding due to its covalent interactions with the molecular target, and it allows for tuning the residence time in the binding site through tailoring the electrophilic group/groups that secure the binding through noncovalent interactions. Below are (Table 5) examples of approved and pending approval RCKIs, as representatives of the cyanoacrylamide class. Over a decade ago, Taunton′s group reported the first RCKI, after evaluating how different substituents around the cyanoacrylamide moiety affect the residence time of the inhibitor [123, 124]. In 2025, the FDA approved the first reversible covalent inhibitor, rilzabrutinib (formerly known as PRN1008), containing the cyanoacrylamide moiety (Table 5). This is a timely addition to the arsenal of covalent kinase inhibitors because its reversibility should reduce the side effects/toxicity due to off‐target binding far less than that of the irreversible inhibitors.

**Table 5:** FDA‐approved reversible Michael acceptors: rilzabrutinib; in clinical trials PRN473 https://pubs.acs.org/doi/10.1021/acs.jmedchem.1c01170.

Structure	Molecular target/activity/MOA	Activity/references
PRN473 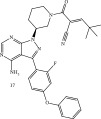	Inhibitor of BTK in B cells; inhibition of IgE (Fc*ε*R)‐mediated activation of mast cells and basophils, IgG (Fc*γ*R)‐mediated activation of monocytes, and neutrophil migration. [120, 121]	In 2025, Completed II (Phase 2a) Study of the Safety, Tolerability, and Pharmacokinetics of Topically Administered PRN473 (SAR444727) in Patients with Mild to Moderate Atopic Dermatitis. [https://clinicaltrials.gov/study/NCT04992546]
Rilzabrutinib. FDA‐approved August 2025 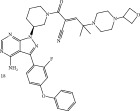	Dual action Inhibitor of BTK in B cells, decreasing macrophage (Fc*γ* receptor)‐mediated platelet destruction and reduced production of pathogenic autoantibodies [122].	Orally available treatment for adults with persistent or chronic immune thrombocytopenia (ITP) who have had an insufficient response to immunoglobulins, anti‐D therapy, or corticosteroids. [https://www.fda.gov/drugs]The drug also has orphan drug designation for the treatment of warm autoimmune hemolytic anemia (wAIHA) and IgG4‐related disease (IgG4‐RD).[https://www.sanofi.com/en/media‐room/press‐releases/2025/2025‐08‐29‐21‐50‐18‐3141825]

**Figure 2:**
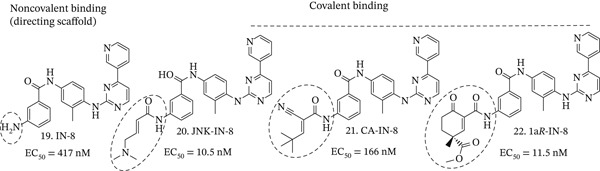
Evolution of c‐jun terminal kinase inhibitor design.

However, the potential of binding of the RCKIs to other molecular targets, as well as cysteins as part of the redox system in the cells, for example, glutathione, still exists. The residence time of the surrounding electrophilic moiety of the inhibitor (e.g., cyanoacrylamide) groups is often evaluated and adjusted through experiments involving binding of the electrophile to thiol groups, using either glutathione or 2‐mercaptoethanol (BME) [123, 124]. In vivo, the reversible inhibitor can be depleted by nucleophiles in the cellular environment that are in millimolar amounts, for example, glutathione. Recently, a step has been taken to “shield” the electrophilic *α*,*β*‐unsaturated system, Compound **21** (Figure 2) to reduce the aforementioned potential interactions with off‐target cysteines [125, 126]. The cyanoacrylamide, as an “open‐chain” electrophile usually easily accessible by a nucleophile, is replaced by cyclic, less nucleophile‐accessible 3D‐shaped Michael acceptor scaffolds, which are found in naturally occurring compounds such as zerumbone [127]. The inherently chiral environment of specific nucleophiles on the protein surface potentially can guide the inhibitors with high specificity to the binding site of the enzyme and diminish its binding to glutathione and other nucleophiles. This strategy, utilizing cyclohexenone‐based Michael acceptor **22** (Figure 2), led to the preparation of inhibitors of c‐Jun terminal kinase that have been tested in the protein–protein interactions of mitogen‐activated protein kinases, such as MAPK D [125, 126]. It was determined that the chirality of the *γ*‐carbon in **1a*R*-In-8** (**22**, Figure 2) plays a significant role in guiding the inhibitor to the “correct” nucleophile and secures binding specificity equivalent to that of its irreversible counterpart **JNK-IN-8** (**20**, Figure 2).

Degrasyn (DGS, WP1130, **23**, Table 6) (not FDA approved), which induces apoptosis in leukemia cells by inhibiting deubiquitination enzymes (DUBs) [132–137], has demonstrated anti‐infective properties. Additionally, the reduction of intracellular replication of *L. monocytogenes* and viruses has been reported. Its anti‐infective properties have been mainly attributed to the inhibition of DUBs in macrophages [138–140]. Direct antibiotic effects of degrasyn, and its derivatives, on isolated bacteria, for example, MDR *S. aureus*, have also been reported [141]. Degrasyn′s antibacterial effect is attributed to several bacterial protein targets as revealed by chemical proteomics. Its molecular targets are important enzyme classes involved in cell wall, lipid and histidine biosynthesis, which demonstrates a polypharmacological mode of action [132]. Recently, the activity of degrasyn against nongrowing and intracellular bacteria has also been reported [133].

**Table 6:** Reversible Michael acceptors with anti‐infective activity.

Structure/main activity (known molecular target)	Molecular target of the “Off‐target” activity/MOA	“Off‐target” activity/references
DegrasynWP1130 (Degrasyn) is a selective molecular deubiquitinase inhibitor and a Bcr/Abl destruction pathway activator that specifically and rapidly down‐regulates both wild‐type and mutant Bcr/Abl protein without affecting bcr/abl gene expression in chronic myelogenous leukemia (CML) cells. 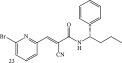	MOA against several important bacterial enzyme classes involved in cell wall, lipid and histidine biosynthesis [132]	Direct antibiotic effects on multiresistant *S*. *aureus* [132], nongrowing and intracellular bacteria, for example, *Escherichia coli* (UPEC). [133]
Cinnamonitriles 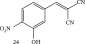 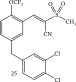	Tyrosine kinase inhibitor. [134] MOA undetermined [135]	Potentiation of oxacillin against *S. aureus*, NRS119 from 256 mg L‐1 to 0.06 mg L‐1 with 3.6 mg L‐1. [135]

A library of cinnamonitriles, represented by Compound **25**, Table 6 [135], which were inspired by a known tyrosine kinase inhibitor **24**, (Table 6) [134] were evaluated as potential adjuvants for restoration of *β*‐lactam antibiotic activity against *S*. *aureus* [135]. Compound **25** (Table 6) demonstrated the best potentiation of oxacillin activity against all MRSA strains tested. Interestingly, despite its structural features, which are based on the structure and activity of its progenitor, kinase inhibitor **24**, Table 6, Compound **25** (Table 6) has no inhibitory activity against the known *S*. *aureus* kinases at a concentration of 100 *μ*M. However, several of the compounds from this library of cinnamonitriles did demonstrate intrinsic antimicrobial activity in the absence of *β*‐lactam antibiotics [135].

## 7. Conclusion

The developments in proteomics recently allowed for profiling of over 1000 compounds as kinase inhibitors and determining the structural basis for the selectivity of one of these compounds as an inhibitor of a mammalian serine/threonine (casein kinase), CK2 [142–144]. But enzyme promiscuity is an important factor in enzyme evolution. It must be noted that typically enzyme modulators rarely act on single molecular targets; therefore, polypharmacology should be an expected outcome. Thus, there are seemingly mutually exclusive consequences in the enzyme modulators′ use and development taking into account that off‐target effects can produce undesirable side effects, whereas synergy between targets for a specific disease can be beneficial. The exploration of the synergy between molecular targets of a given drug/drug candidate requires a deliberate investigation to define the spectrum of that modulator to be performed, a rare undertaking until recently. Perhaps the most profound investigation in identifying the potential of repurposing of clinically relevant drugs was initiated during the SARS‐CoV‐2 pandemic [143]. During that time the design of enzyme inhibitors emerged as a key strategy in drug development, offering potential treatments for a wide range of diseases. Inhibiting specific enzymes can modulate their activity, alter biochemical pathways, and ultimately restore normal physiological functions. However, the design of effective enzyme inhibitors is a complex and multifaceted process that involves various strategies and faces numerous challenges, as many enzymes share structural and functional similarities [1]. A strategy that appears to offer diminished side effects/toxicity, due to reduced residence time on potential off targets, in addition to binding to the targeted enzyme, is the synthesis of reversible covalent enzyme modulators. Covalent binding also enhances the specificity of the modulator toward the intended enzyme. Incorporating a warhead having similar electrophilicity to that of cyanoacrylamide, but carrying a chiral group, further diminishes the potential for off‐target binding, especially to nonenzyme cysteines. We anticipate that more enzymes will be targeted by reversible covalent modulators in the very near future.

## Funding

No funding was received for this manuscript.

## Conflicts of Interest

The authors declare no conflicts of interest.

## Data Availability

The data that support the findings of this study are available from literature sources, which are included within the article.
